# The Potential of Digital Twins in Stroke Care: A Systematic Review of Current Applications and Future Perspectives

**DOI:** 10.34133/csbj.0013

**Published:** 2026-03-31

**Authors:** Jawed Nawabi, Semil Eminovic, Dmitriy Desser, Leonard Fetscher, Ida Rangus, Eberhard Siebert, Mike P. Wattjes, Jana Sofie Weissflog, Aymen Meddeb

**Affiliations:** ^1^ Department of Neuroradiology, Charité—Universitätsmedizin Berlin, Humboldt-Universität zu Berlin, Freie Universität Berlin, Berlin Institute of Health, Berlin, Germany.; ^2^ Department of Radiology, Charité—Universitätsmedizin Berlin, Campus Virchow Klinikum, Humboldt-Universität zu Berlin, Freie Universität Berlin, Berlin Institute of Health, Berlin, Germany.; ^3^ Center for Stroke Research Berlin, Charité-Universitätsmedizin Berlin, Berlin, Germany.

## Abstract

**Background:** Digital twin technology holds promise for personalized stroke care, but current applications remain fragmented. This systematic review investigates how digital twins are currently utilized in the stroke care continuum. **Methods:** Following PRISMA guidelines, we conducted a systematic search of PubMed, Web of Science, and the Cochrane Library through April 2025. Studies applying digital twins to acute ischemic stroke care were included. Each study was categorized along the stroke care continuum (pre-stroke, in-hospital, post-stroke) and assessed using a digital twin maturity framework (L0 to L3). We extracted data on clinical intent, modeling approach, validation strategy, study design, population, sample size, and key outcomes to enable structured synthesis. **Results:** Eight studies met inclusion criteria. Half targeted pre-stroke risk prediction (e.g., modeling atherosclerosis or atrial fibrillation), 2 simulated mechanical thrombectomy, 1 supported prehospital diagnosis, and 1 predicted post-stroke disease progression. Most models remained at maturity levels L1 to L2, lacking real-time updating or workflow integration. Technologies included machine learning (*n* = 3), computational fluid dynamics (*n* = 3), and hybrid or rule-based approaches (*n* = 2). **Conclusions:** Digital twins in stroke care are promising but remain preclinical. Current models predominantly address pre-stroke risk prediction or procedural simulation, with limited representation of acute decision support or post-stroke monitoring. Clinical integration is constrained by low technological maturity, limited real-world validation, and a lack of interoperability.

## Graphical Abstract

**Figure:**
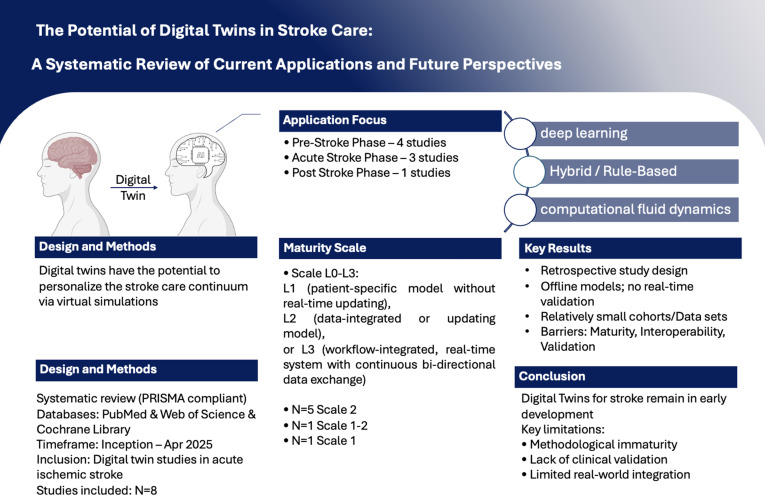

## Introduction

Digital twins are virtual representations of real-world entities that update in real time​. In healthcare, this concept has been applied to monitor patient data and to simulate clinical scenarios​ [1,2]. Stroke remains a leading cause of disability, and despite groundbreaking advances in endovascular thrombectomy, up to two-thirds of large-vessel occlusion stroke patients remain functionally dependent after treatment​ [3]. This has fueled research into novel strategies—including in silico simulations and digital twin models—to personalize stroke care, test therapeutic interventions, and optimize patient selection​. However, using digital twins to predict and improve outcomes in serious conditions like stroke is still an emerging area, with challenges in achieving high accuracy and ensuring data security​ [4]. Therefore, despite the promise of digital twins, their application in stroke care remains sparse and fragmented in the literature. Accordingly, we present a systematic review that synthetized and analyzed clinical studies leveraging digital twin technology in acute stroke care.

## Methods

This systematic review was pre-registered in the International Prospective Register of Systematic Reviews (PROSPERO) [5] under the identifier CRD42024504542 before the start of the initial screening and was conducted according to the Preferred Reporting Items for Systematic Reviews and Meta-Analyzes (PRISMA) guidelines, alongside the PRISMA checklist (Supplementary Materials) [6].

### Definition of a digital twin in stroke care

For the purposes of this review, a digital twin was defined as a dynamic, individualized virtual application that represents a patient, organ system, or clinical care process and is designed to simulate, predict, or inform clinical decision-making [7]. Consistent with prior healthcare digital twin literature, such applications are characterized by the use of patient-specific data, the capacity for simulation or scenario testing, and the potential for iterative updating or prospective interaction with clinical workflows [8]. Studies that employed static simulations, generic population models, or isolated machine-learning (ML) predictors without individualized representation were not considered full digital twins unless they explicitly framed their approach as a patient-specific or care-process-oriented digital twin. These studies were classified as adjacent or preliminary digital twin applications and are discussed separately where relevant.

### Inclusion and exclusion criteria

This systematic review included original studies applying digital twin applications targeting at least one clinical intent relevant to the acute ischemic stroke pathway, including diagnosis, outcome prediction, stroke risk assessment, treatment simulation, clinical decision support, or workflow optimization. Both technical proof-of-concept studies and clinically evaluated models were eligible, as long as they aligned with the definition above. Studies using only simulated or healthy subject data were included only if explicitly designed and validated for future clinical stroke applications. Studies were excluded if their primary focus was on stroke rehabilitation, post-acute stroke care, telemedicine, or long-term recovery, including applications centered on motor function analysis, gait tracking, home monitoring, or post-discharge support. General healthcare studies that referenced digital twins without stroke-specific implementation, as well as studies targeting other neurological conditions, were excluded.

### Review protocol

The search strategy was intentionally clinically oriented and focused on medical databases, as the aim of this review was to evaluate clinical applications and translational readiness rather than computational or engineering methodology development. Therefore, a systematic search was conducted using PubMed, Web of Science, and Cochrane Library to identify relevant studies published in English from the inception of each database through 2025 April 6 [9–11]. The search strategy incorporated Boolean operators, using the terms “Stroke” AND “Digital Twin” OR “Virtual Twin”:((digital twin[Title/Abstract]) OR (virtual twin[Title/Abstract])) AND (stroke[Title/Abstract])) [12]. Articles had to be available in English. Data were extracted using a standardized form that included the following details: author(s), year of publication, publisher, volume and number, year, PMC, and DOI [13,14].

### Screening, data extraction, and synthesis

**Fig. 1.**
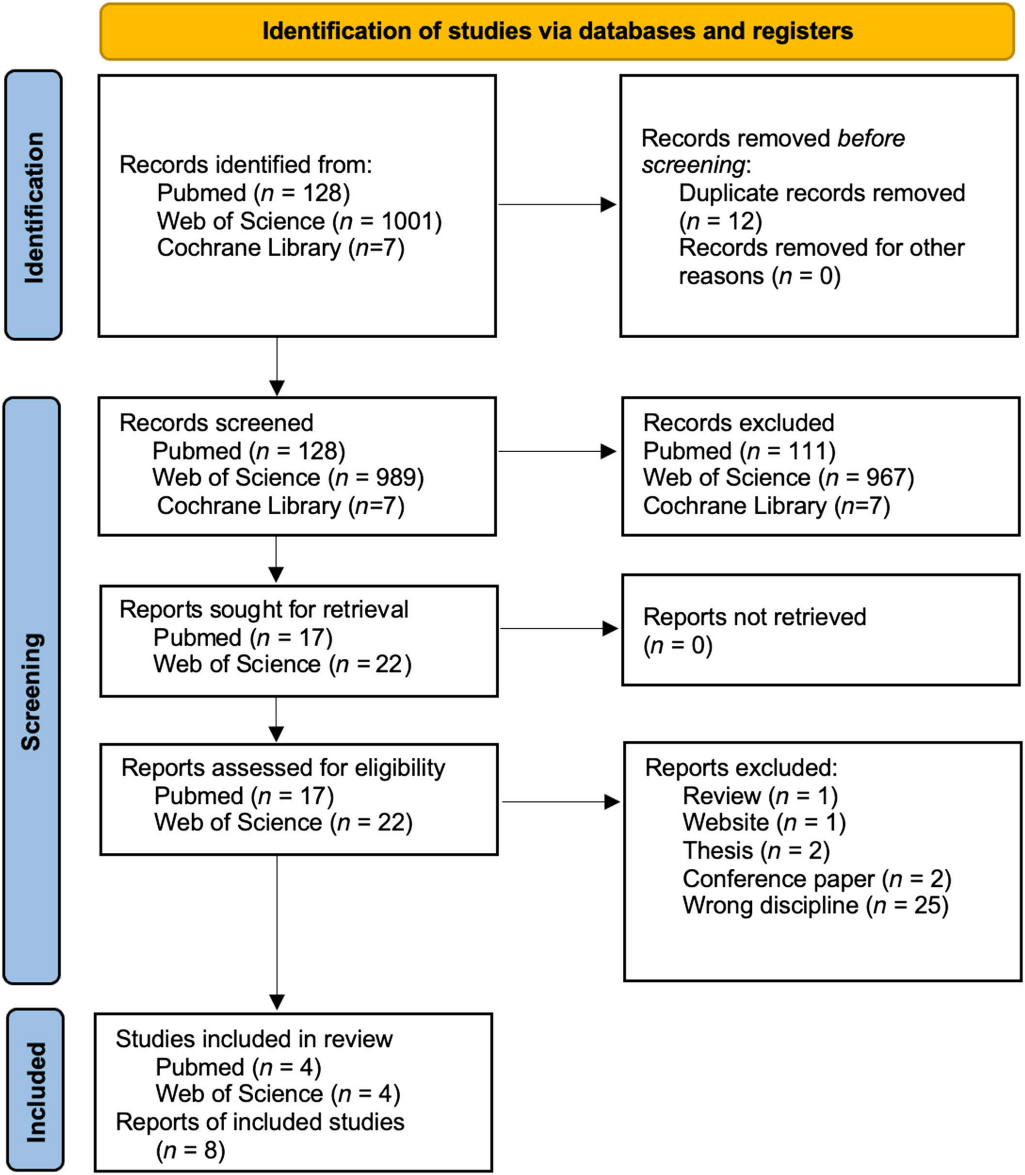
PRISMA flow diagram outlining the study selection process. A total of 1,136 records were identified through database searches (PubMed: 128; Web of Science: 1,001; Cochrane Library: 7). After removing 12 duplicates, 1,124 records were screened by title and abstract. Of these, 1,085 were excluded, and 39 full-text articles were assessed for eligibility. No reports were excluded due to retrieval issues. After full-text screening, 31 articles were excluded for various reasons (review, website, thesis, conference paper, or discipline mismatch). Ultimately, 8 studies met the inclusion criteria and were included in the final qualitative synthesis. Source: Page et al. [6].

Following deduplication, all records retrieved from the 3 databases were screened independently by 2 reviewers (J.N. and J.S.W.) using a 3-step process consisting of title screening, abstract screening, and full-text review (Fig. 1). In the first step, titles were evaluated for relevance to the review objectives, and clearly unrelated studies were excluded. Articles that passed this stage were then assessed based on their abstracts to determine preliminary eligibility. Those identified as potentially relevant proceeded to full-text screening, during which the full inclusion and exclusion criteria were applied. Disagreements at any stage were resolved through discussion and consensus. Studies were included only if a full-text, peer-reviewed manuscript was available. Conference abstracts without full-text versions, preprints, theses, review articles, and non-peer-reviewed sources were excluded at the full-text screening stage [13]. In addition, studies were excluded if they focused solely on unrelated domains such as technology performance, rehabilitation, epidemiology, behavioral health, or telemedicine, or if they described adjacent technologies that did not meet the operational definition of a digital twin. Data extraction was performed using a standardized form that captured key study characteristics including author(s), year of publication, country, study design, population, sample size, clinical intent, primary outcome, outcome measures, and key results [15]. A narrative synthesis was conducted due to the heterogeneity in methodologies and outcomes across the included studies. Each study was categorized along the stroke care continuum (pre-stroke, in-hospital, post-stroke) to thematically analyze the applications and limitations of digital twins in stroke care [16].

### Maturity level classification

To assess the developmental stage of the included digital twin applications, we classified each study according to a maturity framework adapted from prior digital health and simulation research [8,17,18]. This classification considered core functional characteristics including patient specificity, updating frequency, and integration into clinical workflow that were consistent with related, existing maturity taxonomies [19]. Studies were assigned to 1 of 4 levels by 2 reviewers (J.N. and J.S.W.): level 0: conceptual models or frameworks without implementation (e.g., white papers or Delphi mappings), level 1: patient-specific models using static data without real-time updating, level 2: dynamic models capable of simulation or forecasting with individualized data, and level 3: workflow-integrated or closed-loop systems with real-time or prospective validation. These maturity levels are intended as descriptive categories rather than fixed stages of development. A single system may reflect elements of more than one level depending on how it is implemented and integrated into clinical practice.

## Results

### Data extraction and summary of included studies

A total of 8 studies met the inclusion criteria and were analyzed in the main synthesis. Their characteristics, clinical focus, modeling approach, and maturity level are summarized in Tables 1 and 2. The 8 included studies spanned the stroke care continuum, from risk assessment before stroke onset to diagnosis support and modeling of treatment outcomes, and post-stroke clinical progression. The pre-stroke phase was the most represented, with 4 studies (Herrgårdh et al., 2022; Falanga et al., 2024; Upadrista et al., 2025; Dubs et al., 2023) focused on individualized stroke risk prediction [20–23]. These used patient-specific modeling approaches such as atherosclerosis progression (Herrgårdh et al.), left atrial blood flow simulations in atrial fibrillation (AF) (Falanga et al.), blockchain-integrated data streams (Upadrista et al.), and 3-dimensional (3D) hemodynamic modeling of carotid artery stenosis (Dubs et al.). All were classified as L2 or L1 to L2 maturity, indicating real-world data integration but no clinical deployment. In the acute stroke phase, 3 studies explored diagnosis and treatment simulation. Sen et al. developed a prehospital detection tool for arterial occlusion using hemodynamic waveform analysis from wearable Doppler ultrasound, while Bridio et al. (2021) and Fregona et al. (2025) simulated mechanical thrombectomy outcomes based on patient-specific vascular and clot characteristics [24–26]. These studies targeted procedural planning or diagnostic triage, with maturity levels ranging from L1 (static, individualized simulation) to L2 (data-updating models). Only one study, Allen et al., addressed the post-stroke phase, applying deep learning to electronic health records (EHRs) to forecast patient outcomes after an index stroke [27]. This application represents a shift from procedural modeling to longitudinal outcome prediction, with an L2 maturity level reflecting automated, individualized projections without real-time system feedback. Overall, the majority of models (6 of 8) focused on risk or outcome prediction, while real-time decision support and clinical integration (L3) remain absent.

**Table 1.** Characteristics and main findings of studies on digital twin applications in stroke. Synthesis of the study characteristics, methodologies, outcomes, and main findings of 8 studies included in the review.

Title	Authors (year)	Country	Study design	Population	Sample size	Clinical intent	Primary outcome	Outcome measures	Key results	Comments
A digital twins machine learning model for forecasting disease progression in stroke patients	Allen A et al*.* (2021)​ [28]	USA	Retrospective modeling study	Hospital stroke patients (retrospective EHR data)​	1,216 patients (1,094 training + 122 test set)​	Post-stroke clinical progression	Forecasting clinical trajectory and complications	Similarity of simulated data to real data (distribution stats, correlations); adversarial discrimination AUC​ using EHR data	Simulated patient trajectories were statistically indistinguishable from real data​. Adversary could not differentiate real vs. synthetic records (AUC ~0.51)​	Demonstrated a virtual cohort closely mimicking real stroke patient data. Suggests potential for predicting individual trajectories and use as virtual controls in trials​
Impact of the internal carotid artery morphology on in silico stent-retriever thrombectomy outcome	Bridio S et al. (2021)​ [26]	Italy/Netherlands	In silico experiment (with patient CTA data)	Patients with acute ischemic stroke (imaging data from 14 cases)​	14 patient-derived arterial models​	Procedure outcome prediction	Endovascular thrombectomy success	Simulated recanalization success vs. failure; vessel geometric parameters (angles, diameters)	9/14 simulations succeeded vs. 5 failed in clot removal​. Unsuccessful cases had significantly more tortuous vessel anatomy (larger carotid–MCA angles)​	Complex ICA geometry (e.g., sharp angulations) was associated with simulation failure (clot not retrieved)​. Suggests vessel anatomy can influence thrombectomy outcome; findings are preliminary due to small sample.
Digital twins for prediction of atherosclerosis progression and stroke	Herrgårdh T et al. (2022) [21]	Sweden	Computational modeling study	General population (model based on epidemiological/clinical data); no individual clinical implementation yet​	N/A (model development; 1,788 patients mentioned as training dataset in text)	Pre-stroke risk prediction	Long-term stroke risk based on simulated atherosclerosis progression	Model components: risk factor trajectories, plaque buildup, rupture risk, thrombosis risk​; ability to simulate interventions (diet, meds, etc.)	Integrated submodels reproduce known risk factor effects (e.g., dyslipidemia, hypertension, diabetes) on plaque progression and stroke risk​. Model can simulate scenario outcomes (e.g., medication vs. lifestyle changes) for individual risk forecasting​	Demonstrated a proof-of-concept digital twin that can project a patient’s atherosclerosis and stroke risk trajectory​. Not yet clinically tested; planned future validation in preventive health exams​.
Assessment of extracranial carotid artery disease using digital twins—A pilot study	Dubs L et al. (2023)​ [24]	Switzerland	Pilot diagnostic study (cross-sectional)	Patients with carotid artery stenosis (from neurovascular clinic)	37 carotid bifurcations in 32 patients​	Pre-stroke risk prediction	Functional significance of carotid stenosis	CFD-predicted internal carotid peak systolic velocity (PSV) vs. Doppler ultrasound PSV​. Pressure drop under hyperemia in simulations​	CFD-derived velocities agreed closely with ultrasound: mean error ~9%, ICC = 0.88​. Simulated hyperemic conditions revealed marked differences in pressure drop between lesions of similar anatomic severity​	Feasible to create digital twin carotid models from CTA that correlate well with real hemodynamic measurements​. Demonstrated potential to identify functionally significant stenoses via simulation; needs prospective validation.
A digital twin approach for stroke risk assessment in atrial fibrillation patients	Falanga M et al. (2024)​ [22]	Italy	Observational cohort study (cross-sectional analysis)	Non-stroke patients in sinus rhythm: 10 controls, 20 AF (10 paroxysmal, 10 persistent)​	30 patients (with cardiac CT/echo data for LA modeling)​	Pre-stroke risk prediction	Blood stasis and thrombosis risk in atrial fibrillation	LAA and ostium blood flow velocities; endothelial cell activation potential (ECAP, marker of thrombotic risk) in simulations​	AF patients showed significantly lower LAA flow velocities (≈0.04–0.05 m/s) than controls (0.11 m/s)​. . Correspondingly, thrombotic risk index (ECAP) was 4–5× higher in AF vs. controls​	The digital twin simulations revealed hemodynamic differences (stasis and high ECAP) that correlate with known stroke risk in AF​. This approach could refine risk stratification by incorporating patient-specific LA flow dynamics, augmenting clinical risk scores.
A data-driven computational methodology toward a prehospital acute ischemic stroke screening tool using hemodynamics waveforms	Sen A et al. (2024)​ [25]	France/Spain	In silico modeling and ML classification study	Simulated patient data (1D blood flow models for healthy vs. occlusions in different arteries)​	N/A (population-based virtual anatomies; no actual patients)	Stroke detection	Arterial occlusion detection from hemodynamic waveforms	Classification accuracy for occlusion presence and location (in 9 regions) under various noise conditions​	With noise-free data, achieved >95% accuracy in detecting occlusion and identifying its region​. With up to 20% added noise, accuracy dropped to ~80% for region and 70% for vessel-size category​	Demonstrates the feasibility of a digital twin + ML approach for stroke triage using Doppler waveforms​. High performance in ideal settings, though accuracy declines with noise—indicates need for robust sensors and model refinement before real-world use.
Blockchain-enabled digital twin system for brain stroke prediction	Upadrista V et al. (2025)​ [23]	United Kingdom	System development and validation study	General population health dataset (public stroke risk dataset from Kaggle)​; synthetic data augmentation​	4,981 records (Kaggle stroke dataset) + synthetic cases​	Pre-stroke risk prediction	Stroke occurrence prediction	Prediction accuracy for stroke occurrence; system security (blockchain tamper-resistance tests)​	Achieved 98.3% accuracy in predicting stroke risk on the validation dataset​, substantially higher than prior studies (84–92%)​. The consortium blockchain ensured data integrity (tamper-proofing) and privacy​.	A proof-of-concept digital twin platform that can very accurately predict stroke risk in a controlled dataset​. Results likely reflect a curated dataset; authors note need for broader validation due to limited data diversity and static data assumptions​.
Impact of thrombus composition on virtual thrombectomy procedures using human clot analogs mechanical data	Fregona V et al. (2025)​ [27]	Italy/Netherlands	Experimental + simulation study	Lab-created clot analogs (3 composition types: high/medium/low RBC) tested in vitro and in silico​	N/A (no patients; clots from ex vivo analogs)	Therapy modeling	Device–clot interaction by clot composition	Clot mechanical properties (stress–strain behavior) by composition; simulation outcomes: clot fragmentation and retrieval difficulty with different stent devices).	RBC-rich clots were more prone to fragment, whereas RBC-poor (fibrin-rich) clots were harder to retrieve intact​. Higher RBC content led to lower stiffness (softer clots)​. Also, simulation showed sensitivity to thrombus mesh size in the model​.	Demonstrates the feasibility of clot-specific digital twins: mechanical behavior from lab tests was fed into thrombectomy simulations​. Findings align with clinical observations that clot composition affects EVT success. This work can guide device design and personalized thrombectomy strategies, though its preclinical in nature.

AF, atrial fibrillation; AIS, acute ischemic stroke; AUC, area under the curve; CFD, computational fluid dynamics; CTA, computed tomography angiography; DAG, directed acyclic graph; DUS, Doppler ultrasound; ECAP, endothelial cell activation potential; EHR, electronic health record; EVT, endovascular thrombectomy; ICA, internal carotid artery; FFR, fractional flow reserve; GPT, generative pretrained transformer; HPI, history of present illness; ICC, intraclass correlation coefficient; LAA, left atrial appendage; LA, left atrium; LVO, large vessel occlusion; MCA, middle cerebral artery; ML, machine learning; MRI, magnetic resonance imaging; N/A, not available; NCCU, neurocritical care unit; PSV, peak systolic velocity; RBC, red blood cell; SEIPS, Systems Engineering Initiative for Patient Safety

**Table 2.** Studies meeting predefined eligibility criteria for stroke-specific digital twin applications, categorized by clinical focus, aim, and maturity level. Included studies organized by their clinical focus, primary aim, and assigned to a digital twin maturity level. Maturity levels are classified as L1 (patient-specific model without real-time updating), L2 (data-integrated or updating model), or L3 (workflow-integrated, real-time system with continuous bidirectional data exchange).

Authors (year)	Title	Clinical focus	Aim	Maturity level
Herrgårdh et al. (2022) [21]	Digital twins for prediction of atherosclerosis progression and stroke.	Pre-stroke phase	Assessment of stroke risk based on modeling of atherosclerosis progression utilizing multidimensional data	L2
Falanga et al. (2024) [22]	A digital twin approach for stroke risk assessment in atrial fibrillation patients.		Assessment of stroke risk based on atrial fibrillation simulations using a 3D model of the left atrium with CFD simulations	L2
Upadrista et al. (2025) [23]	Blockchain-enabled digital twin system for brain stroke prediction.		A blockchain-integrated digital twin application aimed at predicting the risk of brain stroke utilizing multidimensional data.	L2
Dubs et al. (2023) [24]	Assessment of extracranial carotid artery disease using digital twins—A pilot study.		Assessment of stroke risk based on evaluation of carotid artery stenosis with simulated 3D blood flow models	L1–2
Sen et al. (2024) [25]	A data-driven computational methodology toward a prehospital acute ischemic stroke screening tool using hemodynamics waveforms.	Acute stroke phase	Prehospital diagnosis support system for detection of arterial occlusion with wearable doppler-ultrasound	L1
Bridio et al. (2021) [26]	Impact of the internal carotid artery morphology on in silico stent-retriever thrombectomy outcome.		Treatment outcome related to thrombectomy device and arterial geometry/clot interaction	L1
Fregona et al. (2025) [27]	Impact of thrombus composition on virtual thrombectomy procedures using human clot analogs mechanical data.		Treatment outcome related to thrombectomy device and clot interaction	L2
Allen et al. (2021) [28]	Deep learning model for forecasting disease progression in post stroke patients using EHR data.	Post-stroke phase	Prediction of clinical course after stroke index	L2

3D, 3-dimensional; CFD, computational fluid dynamics EHR, electronic health record

In the following, the included studies are summarized along the stroke care continuum.

#### Pre-stroke phase

##### Herrgårdh et al. (2022)—Digital twins for atherosclerosis and stroke prediction

Herrgårdh et al. introduced a comprehensive digital twin model for atherosclerosis progression, aiming to predict an individual’s future risk of stroke by simulating the development of arterial disease over time​. This work, presented as a conference abstract in *Atherosclerosis*, combines multiple submodels that span from cellular processes up to whole-body physiology. The digital twin takes into account major cardiovascular risk factors, such as cholesterol levels (dyslipidemia), blood pressure, and diabetes, and simulates how they contribute to plaque formation in the carotid arteries, potential plaque rupture, thrombosis, and ultimately stroke risk. By training these submodels on experimental and clinical data and then personalizing them to an individual’s data, the system can generate a trajectory of that person’s arterial health. The authors report that their integrated model can reproduce known patterns: For example, risk factor trajectories influence plaque buildup, which in turn affects stroke risk in a mechanistically plausible way​. An intriguing capability is scenario simulation in which the digital twin can be used to test “what if” interventions like starting a cholesterol-lowering drug or changing diet/exercise, and it will forecast the impact on plaque growth and stroke risk​. The conclusion suggests that this tool will be implemented in a preventive clinical setting (a “health conversation” checkup) to personalize advice and enhance doctor–patient communication​. In summary, this study provides a proof-of-concept that health digital twins can integrate diverse data to predict long-term stroke risk, moving beyond static risk scores to dynamic, personalized modeling.

##### Falanga et al. (2024)—AF stroke risk digital twin

Falanga et al. developed a digital twin of the left atrium (LA) for patients with AF to better assess their individual stroke risk​. In AF, the irregular heart rhythm can lead to blood stasis in the left atrial appendage (LAA) and clot formation, causing embolic strokes. Current stroke risk stratification (CHA₂DS₂-VASc score) in patients with AF is crude and not personalized. This study created patient-specific 3D LA models from cardiac imaging and ran computational fluid dynamics (CFD) simulations of blood flow during sinus rhythm for each patient​. They included 10 healthy controls and 20 AF patients (10 paroxysmal AF, 10 persistent AF)​. The simulations provided detailed measures like blood flow velocity in the LAA and its orifice and a novel metric called endothelial cell activation potential (ECAP), which relates to thrombosis risk on the endocardium​. The findings showed that AF patients’ LAAs had substantially lower flow velocities (around half or less) compared to controls, indicating more blood stasis​. Correspondingly, the ECAP—which can be thought of as an indicator of pro-thrombotic conditions—was substantially higher (4×) in AF patients than in controls​. Patients with persistent AF had slightly worse values than patients with paroxysmal AF, consistent with more remodeling and stasis in persistent AF. These results align with known stroke risks: Reduced LAA flow and more endothelial activation in AF can foster clot formation. The digital twin approach provides a personalized hemodynamic profile, suggesting that in the future, an AF patient’s stroke risk could be assessed by simulating their heart blood flow rather than just counting risk factors. This could identify patients who are at low risk (despite many risk factors) or at high risk (despite few risk factors) based on actual blood flow patterns, potentially guiding anticoagulation decisions more precisely.

##### Upadrista et al. (2025)—Blockchain-enabled digital twin for stroke prediction

Upadrista et al. developed a blockchain-integrated digital twin application aimed at predicting the risk of brain stroke, with an emphasis on high accuracy, data security, and scalability​. The digital twin in this context is essentially a comprehensive virtual profile of an individual’s health, which can be continuously updated and used to predict disease onset. They demonstrate the concept using stroke risk prediction as a case study. The system aggregates data (potentially from medical records, wearables, etc.) and employs an ML model to forecast stroke occurrence. In their reported results, the model achieved an exceptionally high accuracy of 98.28% in predicting strokes on the dataset used​. This is notably higher than typical stroke prediction models (usually in the 80% to 90% range)​. To ensure trustworthiness, they integrated a consortium blockchain system, enabling multiple nodes (e.g., multiple hospitals) to collectively maintain the ledger of digital twin updates, enhancing data integrity and privacy (no single entity can corrupt or expose the data)​. The blockchain ensures that the data feeding the twin are tamper-proof and that access can be audited and controlled health. Their application is also designed to be extensible: While shown for stroke, the authors claim that it can be adapted to other conditions like heart attacks, cancers, etc., by modeling the whole body as needed and plugging in different ML models​. In summary, this study presents a holistic digital twin prototype that combines predictive analytics with cutting-edge data security technology. It achieves a high predictive performance on the chosen dataset, although such high accuracy might partly reflect a controlled environment. The approach nonetheless shows potential as a future health monitoring tool that patients or providers could use to get early warnings of strokes while maintaining strong data governance.

##### Dubs et al. (2023)—Carotid digital twins—pilot study

Dubs et al. conducted a pilot study to assess whether patient-specific digital twins of the carotid artery can improve evaluation of carotid stenosis, which is a risk factor for stroke​. In practice, carotid disease is often graded by the percentage of narrowing and blood flow velocity on Doppler ultrasound, but these do not always predict functional impact (how much blood flow or pressure is actually compromised). The authors created 3D models of 37 carotid bifurcations from patients’ computed tomography angiography (CTA) scans and ran CFD simulations for each—essentially generating a digital twin of the blood flow in each patient’s carotid artery​. They then compared the simulated blood flow metrics to real measurements from Doppler ultrasound for those same patients. They found a high agreement: The CFD-predicted peak systolic velocity in the internal carotid artery (ICA) matched ultrasound values with only ~9% average error and strong correlation [intraclass correlation coefficient (ICC) 0.88]​. Furthermore, they simulated a stress condition (increased flow as would occur during exercise or hyperemia) and observed that some stenotic lesions caused much larger pressure drops under high flow than others, despite similar anatomical narrowing​. This indicates that 2 carotid stenoses that look the same in size may differ in their functional severity—something the digital twin can reveal by “stress-testing” the artery in silico. The study concludes that carotid digital twins are feasible and might serve as a noninvasive tool to assess stenosis-specific stroke risk, analogous to how fractional flow reserve (FFR) is used in cardiology. The authors call this laying the groundwork for future prospective studies to see if CFD-derived metrics better predict outcomes or help decide treatments.

#### Acute stroke phase

##### Sen et al. (2024)—Prehospital stroke screening via digital twin and ML

Sen et al. proposed a novel strategy for rapid stroke diagnosis in the field using a combination of computational modeling and ML​. The aim was to develop a prehospital screening tool to detect and localize arterial occlusions (blockages causing stroke) by analyzing blood flow waveforms, such as those obtainable from a portable Doppler ultrasound device. They created a digital twin of the arterial circulation, specifically a 1D hemodynamic model of blood flow, representing various scenarios: healthy circulation and occlusion in different major arteries (like left or right internal carotid, middle cerebral, anterior cerebral, and posterior cerebral arteries)​. By running simulations on a population-based range of vessel geometries and conditions, they generated a large synthetic dataset of blood velocity waveforms for each scenario. Then, they trained an ML classifier (likely a 2-step classifier) to first detect if an occlusion is present and then identify its general location (e.g., left versus right, and which vascular territory, anterior versus posterior circulation, large versus smaller vessel)​. The results were promising in ideal conditions: over 95% accuracy in both identifying stroke versus no-stroke and roughly pinpointing the occlusion region when using noise-free simulated data​. However, when they added realistic levels of noise (up to 20% noise) to simulate real-world signals, performance dropped to about 80% accuracy for region detection and 70% for finer classification of vessel caliber​. The conclusion is that this approach could be feasible for quick, non-invasive stroke triage [especially distinguishing large vessel occlusions (LVOs) that need thrombectomy], but it would require robust signal processing to handle noisy input. The tool could eventually be deployed on ambulances or in rural clinics as a low-cost stroke screening, essentially acting as a virtual “brain stethoscope” derived from the digital twin’s knowledge.

##### Bridio et al. (2021)—ICA morphology and thrombectomy outcome

Bridio et al. conducted an in silico study examining how the geometry of a patient’s ICA might affect the success of mechanical thrombectomy​. They used imaging data from 14 ischemic stroke patients to create patient-specific arterial models and then simulated a stent-retriever thrombectomy procedure in each model. The digital twin simulation incorporated a realistic clot and a virtual stent retriever device to attempt removal. Outcome was defined as successful clot removal versus failure (clot not extracted or fragments left). The simulation results were then correlated with various geometric measurements of the ICA and middle cerebral artery (MCA) in those patient models, such as vessel diameters, tortuosity, and angulation at the carotid siphon and MCA bifurcation. The key finding was that arterial geometry mattered: Simulations predicted failure in 5 of 14 cases, and those failure cases tended to have ICAs with more extreme curvature and larger angulation between the ICA terminus and MCA branch​. In particular, a higher angle at the carotid T-junction was significantly associated with unsuccessful retrieval attempts. On the other hand, cases with straighter, more favorable anatomy were successfully recanalized in silico. This suggests that certain anatomical factors (like a very tortuous carotid artery) might impede thrombectomy devices from effectively grabbing and pulling clots. The study proposes that patient-specific digital twins could be used pre-procedure to predict difficulty, and that vessel morphology should be considered when planning thrombectomy or designing new devices. However, these conclusions are based on a small sample and simulated outcomes, so they are exploratory.

##### Fregona et al. (2025)—Thrombus composition impact on virtual thrombectomy

Fregona et al. investigated how different blood clot compositions affect the outcome of mechanical thrombectomy (clot removal) using a combination of lab experiments and in silico modeling. They mechanically characterized 3 types of clot analogs with varying proportions of red blood cells (RBCs) and fibrin, representing typical thrombi in ischemic stroke: RBC-rich (red, soft clots), intermediate, and fibrin-rich (white, firm clots)​. These clot analogs were tested to determine their stress–strain behavior and failure thresholds. Then, the authors fed these mechanical properties into a finite element thrombectomy simulation (a digital twin of the clot–device interaction), effectively calibrating the model to each clot type​. They performed virtual thrombectomies using 3 different stent retrievers on clots of each composition to see how often the clot would fragment or resist removal. Key findings include the following: RBC-rich clots are more prone to fragmentation during retrieval​, because they are softer (lower stiffness), whereas fibrin-rich clots are tougher to pull out intact (often stick to vessel or require more force)​. They also discovered the simulation’s sensitivity to technical factors like clot mesh size (numerical artifact)​ representing an important modeling insight. Overall, the study confirms that clot composition significantly influences thrombectomy success: Red, soft clots tend to break apart (risking distal emboli) but are easier to retrieve in pieces, while white, firm clots tend to come out in one piece or not at all (sometimes requiring multiple attempts or different technique). The use of a digital twin allowed them to test multiple clot–device combinations systematically. These results have implications for personalized stroke treatment—e.g., if imaging can infer clot composition pre-thrombectomy, the operator might choose a device or strategy accordingly (aspiration versus stent retriever, etc.), as hinted by the observed differences.

#### Post-stroke phase

##### Allen et al. (2021)—Digital twin ML model for stroke progression

Allen et al. developed an ML powered digital twin model designed to forecast the clinical course of stroke patients using EHR data​. Unlike typical predictive models that output a single risk score or category, this digital twin creates an entire simulated trajectory of the patient’s health data over time​. The authors used a variational autoencoder (VAE), a deep learning approach that encodes input data into a lower-dimensional latent space and reconstructs it, to capture complex patterns in a large retrospective dataset of patients who experienced ischemic stroke. The model was trained on over 1,000 stroke patients’ time-series data (lab values, vital signs, diagnoses) and then tested on an independent cohort​. Key findings show that the simulated patient trajectories were nearly indistinguishable from real patient data: The distribution of vital signs and lab results produced by the digital twins mirrored actual observations, and a classifier tasked with telling real versus synthetic data apart performed no better than chance [area under the curve (AUC) ~0.51]​. This indicates that the digital twin accurately captures the joint patterns in the data. By projecting patient trajectories, the system can, for example, predict the values of labs or likelihood of complications days or weeks before they happen. The authors suggest that such a model could be used to anticipate a stroke patient’s progression under current management or serve as a “virtual control arm” in clinical trials, providing a basis for comparison with interventions​. In summary, this study demonstrates the feasibility of generating realistic virtual stroke patient profiles with ML as an important step toward predictive digital twins in clinical practice.

## Discussion

This review identified 8 digital twin applications across the stroke care continuum. Half of the studies (4 of 8) focused on pre-stroke risk prediction, modeling atherosclerosis progression, AF, or carotid stenosis (e.g., Herrgårdh et al., Falanga et al., Upadrista et al., and Dubs et al.) [20–23]. Two studies (Bridio et al. and Fregona et al.) explored treatment simulation, modeling clot–device interactions during thrombectomy [25,26]. One study (Sen et al.) addressed acute diagnostic support, and another (Allen et al.) modeled post-stroke clinical course prediction [27]. This distribution reflects a skew toward early-phase applications (prediction, simulation), with fewer studies targeting acute decision-making or long-term management.

The granularity of modeling also varied: Some studies simulated organ-level physiology (e.g., left atrial flow and carotid hemodynamics), and others created patient-specific disease trajectories using EHRs (Allen et al.) [27]. A few proposed system-level applications, integrating blockchain or workflow simulations [22]. Four clinical studies did not meet the strict inclusion criteria based on our digital twin definition, but were retained in the supplementary analysis due to their conceptual relevance in an early and heterogeneous field: Konduri et al., Dang et al., Lee et al., and Kolangarakath et al. [28–31] Konduri et al. outlined a foundational vision for stroke-related digital twins, emphasizing integration into neurocritical workflows. Dang et al. applied a Delphi process to derive expert decision rules for stroke care, potentially forming a scaffold for future twin development. Lee et al. explored AI-driven history-taking in neurology, albeit without stroke specificity or a modeling framework. Kolangarakath et al.’s qualitative study used a process simulation to elicit views on prehospital magnetic resonance imaging (MRI) but did not implement a digital twin system. While not meeting inclusion criteria for technical digital twins, this variation underscores the flexibility of the digital twin concept.

Despite their potential, most digital twin applications remained at maturity levels L1 to L2, lacking real-time updating or clinical integration. None achieved L3, which would involve live data feedback and workflow embedding. Digital twin applications were largely tested offline, with no human-in-the-loop trials or deployment in real clinical environments. Even highly accurate simulations (e.g., >95% in silico performance) saw performance drops (to ~80%) under noisy, real-world conditions (e.g., Sen et al.), illustrating a key barrier to translation [24]. In comparison to AI tools already in stroke care, digital twins lag behind [32]. Tools like RAPID or Viz.ai for LVO detection are U.S. Food and Drug Administration (FDA)-cleared and deployed in >1,500 hospitals, offering immediate, interpretable alerts [33]. These deep learning models are typically trained independently on unimodal data (e.g., imaging data) and can be deployed without the need for ongoing adjustment using site-specific data or, if required, only during offline fine-tuning. Digital twins, in contrast, require complex multimodal inputs, real-time synchronization, and robust infrastructures. Real-time data integration is in particular a challenge as hospital data streams are often siloed and not readily connected. Closely linked to this is that the issue of governance and data security remain major barriers [34]. Digital twins rely on longitudinal, multimodal patient data, demanding robust privacy protections. Although blockchain approaches (e.g., Upadrista et al.) offer potential solutions, most studies lacked clear frameworks for data stewardship—an essential prerequisite for clinical deployment [22]. Moreover, these systems introduce technical and operational demands that many hospital information technology (IT) infrastructures are not yet equipped to handle, juxtaposed to deep learning-based models for stroke detection that can be deployed within existing data protection frameworks. Interpretability and trust remain also further key barriers to adoption [35]. Clinicians tend to favor transparent, physiology-based models over opaque ML outputs. In contrast to many FDA-cleared stroke AI tools that deliver simple, actionable predictions, digital twins often involve complex, less interpretable simulations [8]. To address this, some efforts have employed digital twin in the context of neurocritical care using 93 expert-derived rules, arguing that this causal model is more interpretable and credible to clinicians than purely associative ML [29].

While these translational barriers remain substantial, they do not affect all digital twin applications equally. Use cases grounded in established physiological models or image-based simulations, such as the carotid flow and thrombectomy planning twins included in this review, appear particularly well suited for near-term clinical validation, although remaining at a preclinical or early translational stage [23,25,26]. Their localized, single-organ focus facilitates integration into procedural planning or device selection workflows without requiring full-system modeling or real-time data feeds. These focused applications may represent feasible candidates for future prospective validation. Encouragingly, major vendors and startups are becoming involved, indicating increasing translational interest, although clinical implementation remains preliminary. Insights from clot–device simulations [26] or in silico stroke trials [28] could inform the design of commercial neurovascular simulators for training or device testing.

Digital twin technology is also increasingly gaining traction beyond neurology, particularly in cardiology and oncology, where development is more advanced, but many translational challenges remain [19]. In cardiology, digital twins are used to model electrophysiological, hemodynamic, and structural heart behavior, supporting applications like arrhythmia risk stratification and personalized device therapy. Despite this progress, key limitations persist. According to a recent review, external validation of cardiology-related digital twins was found in only 15%, underscoring a major gap [36]. Similarly, uncertainty quantification techniques—such as Bayesian modeling or ensemble predictions—are being explored to improve transparency, yet remain underused [37]. Interoperability challenges are also consistent with the ones we observed in our reviewed studies. Many cardiovascular digital twin prototypes still operate offline, with limited integration into hospital IT systems using standards like HL7 (Health Level Seven) or FHIR (Fast Healthcare Interoperability Resources)—mirroring the challenges observed in stroke applications [38]. Oncology provides another instructive example. Here, digital twins aim to personalize therapy by continuously updating virtual tumor models with genomic, imaging, and treatment data [41]. Leading cancer centers, including MD Anderson, are piloting such approaches to simulate treatment responses in silico [34]. While still experimental, these efforts highlight the potential of digital twins to support complex, individualized treatment planning in real time, and the common hurdles across domains in validation, interoperability, and clinical integration.

This review has some limitations. First, the included studies were highly heterogeneous in scope, methods, and endpoints, precluding a quantitative synthesis and requiring a narrative approach. Second, the boundaries of what constitutes a digital twin in healthcare remain fluid. While we applied minimal inclusion criteria to define digital twins, the evolving nature of the field may still result in differing interpretations. Third, although our systematic search followed PRISMA guidelines and included PubMed, Web of Science, and the Cochrane Library, we did not include additional engineering-focused databases. Finally, we did not conduct backward or forward citation chasing, and gray literature or unpublished industry prototypes were also excluded, potentially omitting some relevant early-stage or proprietary developments.

Taken together, this review found that digital twin applications in stroke care were at an early stage of development, with most models focusing on pre-stroke risk prediction or procedural simulation. While these studies demonstrated conceptual feasibility, all remained in a preclinical stage. Common limitations included a lack of prospective validation, limited interoperability with clinical systems, and minimal human-in-the-loop testing. Compared to other AI solutions in stroke, such as imaging triage tools, digital twins had not yet been translated into routine care. Nonetheless, several reviewed studies demonstrated conceptual feasibility and may represent candidates for future translational evaluation. Based on these findings, future digital twin models should undergo multicenter validation, report standardized performance metrics, and quantify predictive uncertainty. Evaluations need to focus on clinically meaningful outcomes, and systems must align with existing IT infrastructures using interoperable standards such as HL7 FHIR. Usability, explainability, and regulatory readiness remain essential for deployment. As summarized in our proposed “Minimum Requirements for Clinical Translation” (Table 3), meeting these criteria could bridge the current gap between concept and clinical application, providing a structured framework to guide future translational efforts of digital twins in stroke care.

**Table 3.** A minimum reporting set for digital twin studies in stroke care. This table outlines key reporting criteria to enhance transparency, reproducibility, and clinical readiness of digital twin models in stroke care.

Category	Description
Clinical intent and use case	Define the purpose of the digital twin (e.g., stroke risk prediction, diagnosis, treatment simulation). Specify the target level (organ-specific, patient-level, or population).
Model granularity	Indicate the scale of the model: organ-level (e.g., carotid artery), patient-level (e.g., disease trajectory), or system-level (e.g., workflow modeling). Clarify scope and level of detail.
Patient-specific initialization	Detail how the twin is individualized, including input data types (e.g., imaging, hemodynamic data, clinical records).
Update behavior and latency	Describe update type (static, episodic, dynamic), frequency, and data latency.
Modeling architecture	Indicate whether the model uses mechanistic, data-driven (e.g., ML), or hybrid methods. Describe architecture briefly.
Validation and testing	List validation methods (e.g., cross-validation, external validation, prospective studies). Clarify clinical relevance.
Uncertainty and robustness	Explain how model uncertainty is quantified or handled (e.g., sensitivity analysis, probabilistic outputs).
Intended decision impact	Describe the clinical role (e.g., predictive tool, decision support) and whether usability was tested with users.
Data security and interoperability	Describe measures for data protection and compliance, and use of interoperability standards (e.g., HL7/FHIR, blockchain).

HER, electronic health record; HL7, Health Level Seven (international interoperability standard for health information); FHIR, Fast Healthcare Interoperability Resources (a standard describing data formats for electronic health records); ML, machine learning

## Data Availability

All the articles and data sources reviewed in this systematic review are publicly available online. Readers can access the full-text articles and associated data through the respective journal websites, databases, or repositories where they were originally published or hosted.
